# Tartrite of Antimony a Cure for Hernia Humeralis without Bleeding

**Published:** 1816-10

**Authors:** Francis Bush

**Affiliations:** Member of the Royal College of Surgeons, &c. &c.


*For the London Medical and Physical Journal.*

*Tartrite of Antimony a Cure for Hernia Humeralis without Bleeding;* by FRANCIS BUSH, Member of the Royal College of Surgeons, &c. &c.

MR. CLARK's Case of Inflamed Testis, published in your last Journal, induces me to offer a few remarks on that disease, and to suggest a plan of cure I have uniformly pursued with the most complete success for the last ten years.

Hernia humeralis is well known to arise most commonly from the use of injections, especially of lead, for the cure of gonorrhœa; but it is not unfrequent where no injection has been used. At an early period of my practice, I treated this disease by general and topical bleeding, and refrigerant lotions; and, though I do not remember to have carried blood-letting to such an extent as your correspondent, Mr. Clark, did, in the case he has detailed, I was, in many cases, obliged to reduce the system considerably, before I could effect the revolution of the disease. Finding that this plan of cure was tedious, and effected by great violence done to the powers of life, I was induced to try the effect of Tartrite of Antimony, given in doses twice a-day, so as to produce nausea, or even vomiting. My first attempt succeeded to effect a cure in a few days; and every succeeding case has given way to the same plan, so that for the last ten years I have not used any other remedy. I have met with no rebellious case,—not one in which bleeding, topically or generally, has been resorted to.

*Frome; Sept.* 5, 1816.

